# Liposomal gp100 vaccine combined with CpG ODN sensitizes established B16F10 melanoma tumors to anti PD-1 therapy

**DOI:** 10.22038/ijbms.2020.46654.10762

**Published:** 2020-08

**Authors:** Mona Yazdani, Mahdi Hatamipour, Behrang Alani, Hossein Nikzad, Nema Mohamadian Roshan, Javad Verdi, Mahmoud Reza Jaafari, Mahdi Noureddini, Ali Badiee

**Affiliations:** 1 Department of Applied Cell Sciences, Faculty of Medicine, Kashan University of Medical Sciences, Kashan, Iran; 2 Nanotechnology Research Center, Pharmaceutical Technology Institute, Mashhad University of Medical Sciences, Mashhad, Iran; 3 Anatomical Sciences Research Center, Faculty of Medicine, Kashan University of Medical Sciences, Kashan, Iran; 4 Department of Pathology, School of Medicine, Mashhad University of Medical Sciences, Mashhad, Iran; 5 Department of Tissue Engineering and Applied Cell Sciences, School of Advanced Technologies in Medicine, Tehran University of Medical Sciences, Tehran, Iran; 6 Biotechnology Research Center, Pharmaceutical Technology Institute, Mashhad University of Medical Sciences, Mashhad, Iran; 7 Department of Pharmaceutical Nanotechnology, School of Pharmacy, Mashhad University of Medical Sciences, Mashhad, Iran

**Keywords:** Anti PD-1 monoclonal-antibody, CpG ODN, GP100, Liposome, Melanoma

## Abstract

**Objective(s):**

Program death 1 (PD-1)/ program death-ligand 1 (PD-L1) pathways, as the main inhibitory checkpoints, induce immunosuppression in the tumor microenvironment (TME). Despite the importance of inhibitor checkpoint receptor (ICR) blockers, their outcomes have been limited by the low immune response rate and induced acquired resistance. Pre-existing tumor-specific T cells is related to the improvement of their therapeutic efficacy. In the present study, we show that the combination of liposomal gp100 nanovaccine with anti PD-1 monoclonal antibody (mAb) potentiates the therapeutic effect in the melanoma model.

**Materials and Methods:**

In this study, we first decorate the cationic liposome with gp100_25-33_ self-antigen and then characterize it. Mice bearing B16F10 melanoma tumors were vaccinated with different formulations of gp100 peptide (free or liposomal form) with or without CpG ODN adjuvant in combination with anti PD-1 mAb.

**Results:**

Therapeutic combination of liposomal nanovaccine and CpG with anti PD-1 mAb, demonstrated the increased number of tumor infiltrated lymphocytes (TILs) in TME with the highest IFN-γ production and cytotoxic activity, which led to remarkable tumor regression.

**Conclusion:**

Our results demonstrated the synergism between Lip-peptide+CpG nanovaccine and anti PD-1 regime, which improved the therapeutic efficacy of PD-1 checkpoint blocker in melanoma mice models.

## Introduction

During the past decades, various cancer immunotherapeutic approaches have been discovered and evaluated against different types of cancers such as *ex vivo* generated dendritic cell-based (DC) vaccines, engineered T cells, and inhibitory checkpoint receptor (ICR) blockers (1). 

The immune-suppressive characteristics of the tumor microenvironment (TME) mediated *via* the dynamic expression of co-inhibitory molecules and cytokines by immunosuppressive cells, is the main evidence which influences the success of cancer immunotherapeutic strategies (2). Program death 1 (PD-1) is one of the major co-inhibitory checkpoints in the induction of immune suppression through binding of PD-1 receptor on activated T cells to its ligand, PD-L1, and on tumor cells, which significantly inhibit the killing activity of cytotoxic T lymphocytes (CTLs) (3). Despite the importance of ICR blockers such as anti PD-1 monoclonal antibodies (mAb), which are considered as their benefits, their results have been disappointing due to the induced immune resistance when applied as a monotherapy (4, 5). It is stated that the favorable therapeutic result of anti PD-1 blockers is related to pre-existing tumor-specific CTLs (2, 6). In this regard, PD-1 blockers have been used in combination with cancer vaccines (7). It was shown that the combination of cancer vaccines and PD-1 blockades had therapeutic benefits even for less immunogenic tumors such as melanoma (7). 

Therapeutic cancer vaccines have been considered to expand the pool of anti-tumor specific T cells besides reactivation of pre-existing anti-tumor anergic T cells, enhancing the infiltration of T cells into TME as a consequence (2). In this regard, enhanced presentation of tumor-associated antigens (TAAs) to antigen presenting cells (APCs) like DCs, co-administration of immunomodulators and antigens to the same DCs and persistent activation of tumor-specific T cells are the essentials of therapeutic cancer vaccine efficiency (8, 9). 

Liposomes have been regarded as a promising antigen delivery system in the field of cancer vaccine owing to their ability to co-deliver Ag and adjuvants, protecting TAAs from degradation and efficient uptake by DCs (10, 11). Liposomes’ size and surface charge as physicochemical characteristics have a vital role in recognition and uptake by DCs (12, 13). Among different types of liposomes, cationic ones are well-known delivery systems improving the immunogenicity of poorly immunogenic antigens (14). Besides that, DOTAP as a cationic lipid itself, has an immunomodulatory property, stimulating the expression of DCs co-stimulatory molecules (CD80, CD86), activating DCs maturation *via* triggering MAPK pathway, which leads to the production of T helper type 1 (Th1) related cytokines such as IL-12 and eliciting CD_8_^+^ T cells (15, 16). 

To design an effective cancer vaccine, co-administration of an immunomodulator with antigen is of crucial importance (17). Toll-like receptors (TLRs) agonists have been widely used as an adjuvant alongside therapeutic cancer vaccines (18, 19). Their adjuvanticity is mainly attributed to the induction of innate immune responses, but co-delivery of Ag to DCs will enhance the cross-presentation of Ag and leads to the activation of adaptive immune responses (20). Since TLR9 is predominantly expressed in DCs, CpG-ODN, as an agonist of TLR9, induce Th1 and tumor-specific CTLs *via *endosome uptake and maturation of DCs (21, 22) as well as the secretion of IL-12 cytokine (23). It was revealed that co-administration of the electrostatic interacted complex of CpG-ODN and cationic liposomes consisting of DOTAP has improved the immune responses with a prolonged protective period (24). On the other hand, apart from standard immunogenic features of CpG-ODN, it was found in preclinical experiments that incorporation of CpG-ODN in therapeutic strategies could support anti-ICR immunotherapeutic effects (25). The Combination of CpG-ODN with anti PD-1 mAb regime has resulted in prolonged survival time (26) and increased response to anti PD-1 treatment with the generation of durable T cell responses (27). 

Based on the presented observations, we have synthesized the covalently linked melanoma antigen, gp100, with cationic liposomes, as a cancer vaccine co-administered with CpG-ODN for *in vivo* delivery to DCs to improve the immunotherapeutic effect of anti PD-1 therapy. The therapeutic efficacy of this combination was then evaluated in melanoma tumor-bearing C57BL/6 mice. 

## Materials and Methods

***Materials***

N- [1-(2, 3-Dioleoyloxy) propyl]-N, N, N-trimethylammoniummethyl-sulfate (DOTAP) and 1, 2-distearoyl-Sn-glycero-3-phosphoethanolamine-N- [maleimide (polyethylene glycol)-2000] (DSPE-PEG2000-Maleimide) were obtained from Avanti Polar Lipid (Alabaster, AL, USA). Cholesterol, Lipopolysaccharides (LPS) and Hyaluronidase enzyme were obtained from Sigma-Aldrich (Steinheim, Germany). Recombinant Mouse GM-CSF and IL-4 were obtained from Biolegend (San Diego, USA). 1,1-Dioctadecyl-3,3,3,3-tetramethylindodicarbocyanine (DiD), Phytohemagglutinin (PHA), Calcein AM (AM=acetoxymethyl), were obtained from Invitrogen (Carlsbad, CA). All the flow cytometry antibodies and kits were obtained from BD Biosciences (San Diego, USA). Collagenase Type I enzyme was obtained from Gibco (UK). Anti-mouse PD-1 (CD279) monoclonal antibody was obtained from BioXCell (West Lebanon, USA). All other solvents and reagents were used as a chemical grade.

***Peptide and oligonucleotide***

The synthesis of the murine modified gp100_25-33_ peptide containing linker sequence (Ac-CGGGEGPRNQDWL) and purification (95%) was performed by China Peptides Co (Shanghai, China). The synthesis of CpG-ODN 1826 class B (5’-tccatgacgttcctgacgtt-3’) with phosphorothioated backbone was performed by Bioneer Co (Korea).

***Tumor cell lines and animals***

The B16F10 murine melanoma cell line derived from C57BL/6J mice, which express the gp100 antigen and the CT26 murine colon carcinoma cell line, were obtained from the Pasteur Institute (Tehran, Iran), each cultured respectively in 10% FBS containing DMEM and RPMI-1640 media.

6 to 8-week old female C57BL/6 mice were obtained from the Royan Institute (Tehran, Iran). Animal procedures were conducted in accordance with guidelines approved by the Ethical Committee and Research Advisory Committee of Mashhad University of Medical Sciences.

***Conjugation of GP100 peptide to DSPE PEG2000 maleimide***

The mixture of gp100 peptide with DSPE-PEG2000-Maleimide (DMSO: Chloroform 1:1) at 1.2:1 molar ratio was incubated and stirred for 48 hours at room temperature (RT) in order to fabricate covalent conjugates *via* attaching the pyrrole group to the thiol group of cysteine amino acid of maleimide and the linker sequence of the peptide. Following the incubation time, the linkage of the final product was checked by the thin-layer chromatography (TLC) method followed by the evaporation of the solvents (DMSO and chloroform) using a rotary evaporator and freeze dryer. The obtained powder re-dissolved in sodium chloride (NaCl), which was then analyzed with High-performance liquid chromatography (HPLC) quantifying the percentage of conjugation by determining the unconjugated amount of peptide. A Nucleosil C18 column (5µm, 150×4.6mm, 100Aº column, Knauer, Germany) was eluted with the mobile phase composed of water and acetonitrile each containing 0.1% TFA (30:70). The flow rate was 1ml/min and the UV detection wavelength was set at 220 nm.

***Synthesis of liposomal formulation and characterization***

Liposome was formulated using the lipid film hydration method. In brief, the prepared lipid film consisted of DOTAP: Cholesterol with total lipid concentration of 8mM (5:3 molar ratio) was hydrated with HEPES buffer (10mM, pH 7.4) containing Sucrose (9.5 %w/v), sonicated and extruded *via* 200 and 100 nm polycarbonate filters in order to form vesicles with ~100 nm diameters. Finally, the post-insertion of conjugated peptide (GP100-mPEG-DSPE) micelles into the liposome formulation was carried out by incubation for 2 hr at 55 ^°^C. The final liposomal formulations (with or without CpG) were analyzed for size, polydispersity index (PDI) and zeta potential *via* the dynamic light scattering (DLS) instrument (Malvern, UK). For liposomal formulation with CpG, liposome construct and CpG were incubated 30 min prior to analysis, the same was applied for Peptide+CpG group. 

***The ability of cationic liposomes and peptide in the induction of BMDC maturation***

BMDCs generated from mouse hematopoietic progenitor cells reside in bone marrow according to the protocol used in previous studies (28, 29). Immature BMDCs were incubated with an optimum, non-toxic dose of cationic liposomes (12 nmol/ml) (30) and gp100 peptide (2.5 μg/ml) for 8 h at 37 ^°^C, the same time used for the control group stimulated with LPS (1 μg/ml). Following the incubation time, cells were harvested and marked for DCs specific CD markers (CD11c, CD80, CD86, CD40, MHC II). The expression level of co-stimulatory molecules was then assayed using the FACS Calibur flow cytometer (BD Biosciences). 

***In vivo tracking of liposomal formulation***

To study lymphoid distribution, mice were injected subcutaneously (SC) in the groin with DiD-labeled (5µM) nanoliposomes and tracked post-injection, at different time points. DiD-labeled cationic liposomes were prepared using the lipid film method as was mentioned above and post-inserted with GP100-mPEG-DSPE. 100 µl of labeled liposomes were injected and the fluorescence was tracked with the excitation at 644 and emission at 665nm using Kodak *in vivo* imaging system F pro (USA). Results were analyzed with Kodak molecular imaging software version 5 (Science park west, New Haven, CT, USA) and presented as the fluorescence intensity at the injection site and lymph nodes at different time points.

***In vivo therapeutic schedules of liposomal vaccine and Anti PD-1 mAb***

B16F10 tumors were induced in C57BL/6 mice by *SC* injection (3×10^5^ cells/mouse) into the right flank. On day 10 of post tumor transplantation, when the tumor size reached 0.5 cm (31), mice were divided randomly into five groups (n=10) and received the priming dose of vaccines *SC* in the groin followed by 2 booster doses on days 17 and 24. The treatment groups were as follows: Buffer (as control), peptide (alone), Lip-peptide, Peptide+CpG and Lip-peptide+CpG. All groups received 50µg/100µl of the peptide in either form of free or liposomal. For two other groups consisting of CpG ODN, peptide or liposomal form were mixed with CpG ODN and left for 30 mins at room temperature before administration (0.002 µmol of CpG ODN per 0.24 µmol of total lipid) (Table 1). All mice received combination therapy by IP administration of 4 doses of the anti-PD-1 mAb (100 µg/100µl PBS) (32) with 3-day intervals starting on day 11, repeated on days 15, 19 and 23 (33, 34). Tumor volumes and body weight were measured every 2 days during the experiments. Tumor volume was calculated according to the formulae as length×width×height×Л/6 (35). The therapeutic efficacy in treated mice was followed for 25 days and mice were excluded from the experiment when the tumor volume exceeded 1000 mm^3^ or mice were ulcerated (36, 37). TTE, TGD, MST and ILS for each group were calculated (38, 39). On day 26, the experiment was terminated and three mice from each group were sacrificed for further analysis. It was ensured that the number of animals in each group was sufficient for statistical analysis and obtaining significant data.

***Splenocyte and tumor single-cell suspension***

Splenocyte and tumor single-cell suspensions were prepared from dissected spleen and tumor tissues of experimental animals initially by mechanical disruption followed by enzymatic digestion of tumors with Collagenase type I (2mg/ml) and Hyaluronidase (1mg/ml) for 2 hr at 37 ^°^C – not for spleens – and afterward, passing through 70 µm cell strainer and lysis of RBCs with ACK lysis buffer. The obtained single cells were counted and used for further immunological analysis. 

***Flow cytometric assessment of antitumor-specific immune responses***

The obtained spleens and tumors single cells at a density of 10^6^ cells/wells were re-activated with 10µg of gp100 peptide and also treated with brefeldin A. After 24 h, the activated cells were collected and divided into different flow cytometry tubes for extra and intracellular staining to analyze the levels of cell surface markers and cytokines production of CTL, Th and regulatory T cells (Treg) using FACS Calibur flowcytometer (BD Biosciences). The staining procedure was performed according to the manufacturer’s instructions.

***Functional analysis of T cells via Enzyme-linked Immunospot (ELISpot) assay***

Splenocyte and tumor cells were seeded in IFN-γ antibody pre-coated wells to evaluate IFN-γ production. Peptide wells containing 3×10^5 ^cells were activated with 10µg/ml peptide for 24 hours. The secreted IFN-γ was detected by adding a detection antibody followed by streptavidin-HRP and finally, the blue precipitate spots were visualized by adding TMB substrate solution. PHA (10µg/ml) and media wells containing 10^5 ^cells were considered as positive and negative controls. The visualized spots were counted with Kodak 1D image analysis software (Version 3.5, Eastman Kodak, Rochester, New York, USA). The normalized results estimated as Mean±SD in triplicate wells were expressed as spot-forming units (SFU) per 10^6 ^cells. All the procedure was carried out according to the manufacturer’s protocol of mouse IFN-γ basic ELISpot kit (Mabtech, Sweden).

***In vitro activity of Tc cells (CTL)***

The antitumor activity of splenocytes from treated mice was evaluated against the B16F10 cell line. Briefly, B16F10 cells labeled with Calcein AM (12.5 µM) (2×10^4 ^cells per well), served as target cells (T), were seeded in triplicate wells and co-cultured with splenocytes, served as an effector cell (E), at E:T ratios of 20/1, and 40/1. After a 4 hr incubation, the killing activity of CTL was evaluated by analyzing the fluorescence of supernatant using a fluorescent plate reader with excitation at 485 nm and emission at 538 nm (FLx800, BioTek Instruments Inc. USA). The percentage of specific lysis was calculated as (release of effector cells-minimum release)/(maximum-minimum release)×100. The target cells treated with media containing 2% Triton X-100 and just media were served as a maximum and minimum release, respectively.

***mRNA expression level analysis via quantitative real-time PCR***

Liquid nitrogen-frozen spleen and tumor tissues of treated mice were used for extraction of total RNA using the Column RNA isolation kit (Denazist, Iran) according to the manufacturer’s instructions, and their concentration was measured using NanoDrop one^c^ (Thermo scientific, USA). Then, total RNA was used to transcribe into cDNA according to the manufacturer’s guidelines of cDNA synthesis kit (Yekta Tajhiz Azma, Iran). The mRNA expression levels of IFN-γ, IL-10 and PD-1 were quantified using a two-step Sybr Green real-time PCR kit (Yekta Tajhiz Azma, Iran) and normalized relative to the housekeeping gene, GAPDH. The real-time PCR was run in triplicate with 100ng of cDNA using the following primers: IFN-γ (Forward: 5’GCTCTGAGACAATGAACGCT3’, Reverse: 5’AAAGAGATAATCTGGCTCTGC3’), IL-10 (Forward: 5’TGAGAACAGCTGCACCCACT3’, Reverse: 5’GGAAACCCAGGTAACCCTTA3’), PD-1 (Forward: 5’TTTCAGGAATGGGTTCCAAG3’, Reverse: 5’ACATCCTACGGTCCCAAGGT3’) and GAPDH (Forward: 5’TGCACCACCAACTGCTTAG3’, Reverse: 5’GATGCAGGGATGATGTTC3’) (40, 41). The Rotor gene Q instrument (QIAGEN Hilden, Germany) was used with the running program described by the manufacturer as: 3min at 95 ^°^C, followed by 40 cycles 20 sec at 95 ^°^C, 20 sec at 60 ^°^C and 5 sec at 72 ^°^C.

***Immunohistochemistry assay***

Formalin-fixed paraffin-embedded (FFPE) sections of melanoma tumor samples with 5μm thickness were mounted on activated slides and in order to retrieve antigen epitopes, were first deparaffinized and then retrieved using antigen retrieval 10x for 35sec at 98 ^°^C. Following that, the sections were incubated by H_2_O_2_ 5% for 10sec at RT. Afterward, the samples were incubated with 1/400 concentration of anti PD-1 primary antibody for 15 min in dark. The Mouse/Rabbit PolyVue Plus™ HRP/DAB Detection System kit (Diagnostic Bio Systems, Pleasanton, CA, USA) was used to visualize the samples, and for the final step, tumor sections were also stained for morphological characterization with Hematoxylin. 

***Statistical analysis methods***

The results were analyzed with a one and two-way analysis of variance, followed by Tukey post hoc test using GraphPad Prism software (Version 8) to compare the difference between multiple groups. The statistical significance of survival was determined with a log-rank (Mantel-cox) test. *P*<0.05 was served as statistically significant. 

## Results

***Characterization of gp100-mPEG2000-DSPE conjugation***

**Figure 1:**
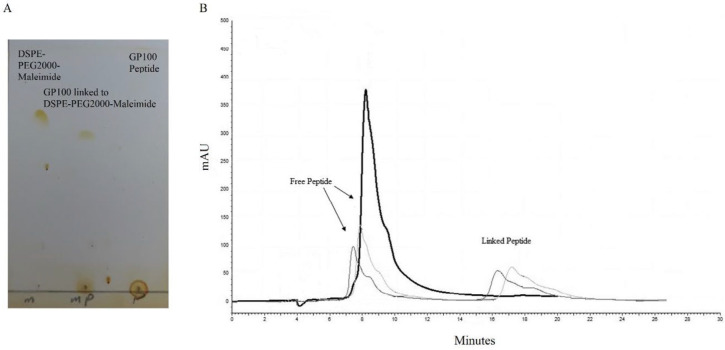
Confirmation of gp100 peptide conjugation to Maleimide-PEG2000-DSPE via (A) TLC and (B) HPLC methods

GP100-mPEG-DSPE micelles were prepared by conjugating gp100 peptide to DSPE-PEG2000-Maleimide through covalent binding. The success of conjugation was confirmed *via* the TLC method by fading the final product solvent spot on silica gel. The percentage of conjugation was estimated according to the area ratio of unconjugated peptide peak *via* HPLC analysis with a yield of 70% (Figure 1A & B).

***Characterization of liposomal formulations***

As shown in Table 2, the diameter of Lip-peptide formulation was increased due to the presence of PEG following post-insertion. The Addition of CpG ODN to the cationic liposome and incubation prior to injection had increased the diameter of formulation which was attributed to the electrostatic interaction between the positive charge of DOTAP containing liposome and negative charge of CpG ODN, which indicated the proper absorption of adjuvant to the liposomal formulation. Interestingly, incubation of CpG ODN with gp100 peptide led to nanoparticle formation *via* electrostatic interaction with a desirable size (166 nm) for uptake by DCs. By considering zeta potential results, in the Lip-peptide group, the presence of peptides and PEG on the surface of the liposomes decreased the charge of DOTAP containing formulation. For liposomal formulation with CpG ODN, the addition of CpG besides the presence of peptide and PEG, notably decreased the charge of the final product. 

Table 2 shows the lipid dose, peptide and CpG ODN that each mouse received per injection. The dose of CpG for Peptide+CpG group was the same as the liposomal group, as was determined according to the dose of DOTAP in each injection.

***Both peptide and liposome promoted BMDCs maturation***

**Figure 2:**
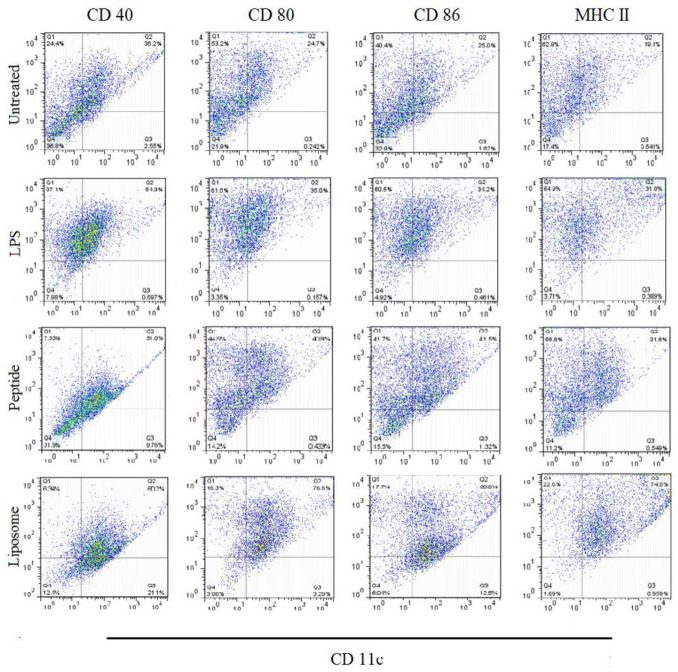
Flow cytometric analysis of BMDCs maturation

We evaluated the effects of cationic liposome and gp100 peptide on the maturation of BMDCs *in vitro* by measuring the expression levels of the co-stimulatory molecules CD40, CD80, CD86, and MHC II receptor that indicate the BMDC maturation. As shown in Figure 2, gp100 peptide could enhance the expression levels of all three co-stimulators similar to LPS treatment. Results from treatment with cationic liposomes showed significantly higher expression levels than treatment with LPS. Altogether, the presented results confirmed the immunological potency of both candidate peptide and cationic liposomes in the induction of DCs maturation. 

***Trafficking of fluorescent-labeled liposomes to the tumor-draining lymph nodes (TDLN)***

**Figure 3:**
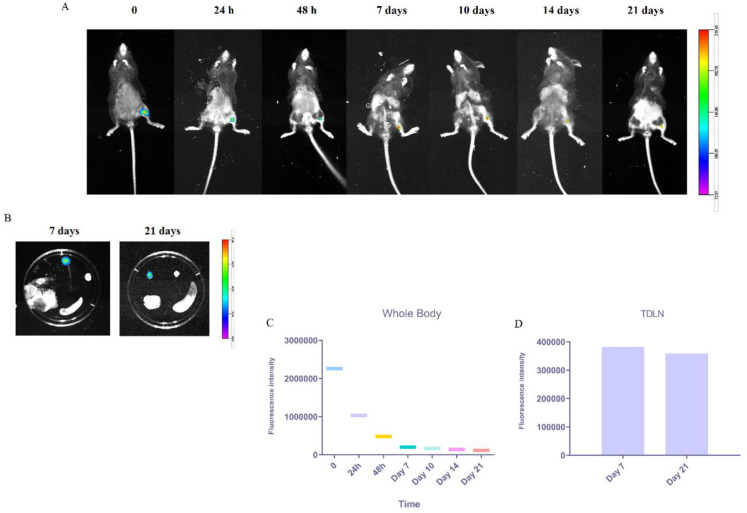
*In vivo* lymphatic trafficking of s.c. administrated nanovaccine by animal imaging

We injected SC DiD-loaded liposomes in the groin of mice to investigate the trafficking of liposomal peptide vaccine to tumor-draining lymph nodes (TDLN) and tracked them *in vivo* at different time points. As shown in Figure 3A & C, strong fluorescence signals were detected in the injection site right after injection and 24 hr then obviously decreased at 48 hr followed by gradual disappearance from 7 to 21 days post-injection. On days 7 and 21, post-injection labeled liposomes were still abundant in the TDLN (Figure 3B & D). All the data can confirm the successful uptake by resident DCs and their migration from the injection site toward the lymphatic sites.

***Enhanced immune responses by combination therapy***

*The Frequency of T cell subpopulation induced by therapeutic vaccines*

**Figure 4:**
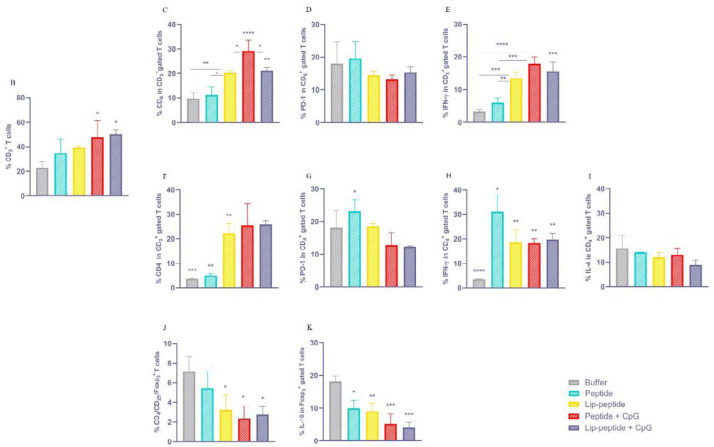
Immunogenicity of liposomal nanovaccine

Flow cytometric analysis of antigen-specific immune responses has revealed that combination therapy with peptide+CpG or Lip-peptide+CpG vaccines have significantly (*P*<0.01) increased T cell population compared to the control group (just anti PD-1 therapy) (Figure 4B). By considering CD_8_^+ ^T cells, both liposomal formulations, with or without CpG, and peptide+CpG groups have stimulated potent antigen-specific CD_8_^+ ^T cell responses and IFN-γ CD_8_ double-positive T cells could be detected significantly in the spleen of treated mice. As shown in Figure 4C, treatment with a liposomal form of gp100 peptide significantly expanded antigen-specific CD_8_^+ ^T cells compared to peptide or control groups (*P*<0.05 and *P*<0.01). In the peptide+CpG group, a significant (*P*<0.05) increased response was shown over Lip-peptide or Lip-peptide+CpG groups and over peptide or control groups. The Lip-peptide+CpG group also showed a significant difference compared to peptide or control groups (*P*<0.01). Results of IFN-γ in CD_8_^+^T cells (Figure 4E) have shown that Lip-peptide, Lip-peptide+CpG, and peptide+CpG groups had a significant difference (*P*<0.001 and *P*<0.0001, respectively) in comparison with the control group. Also, compared to the peptide group, peptide+CpG or Lip-peptide+CpG groups significantly increased IFN-γ secretion with *P*<0.001 while Lip-peptide group did so with *P*<0.01. In the results of the CD_4_^+ ^T cell population (Figure 4F), treatment with a liposomal form of gp100 peptide markedly increased the number of antigen-specific CD_4_^+^T cells over peptide and control groups (*P*<0.001). Both formulations consisting of the CpG adjuvant had a significant difference with peptide (*P*<0.01) or control (*P*<0.001) groups, as were detected in spleen. The Analysis of IFN-γ secretion by Th1 CD_4_^+^ T cells showed that the peptide group had the highest titer of cytokine compared to buffer (*P*<0.0001) or other groups (*P*<0.05). A Significant difference was also detected for other groups compared with control groups (*P*<0.01). In contrast to IFN-γ, the analysis of IL-4 secretion by Th2 CD_4_^+^ T cells showed no significant difference between vaccinated groups in the spleen (Figure 4H & I). The Analysis of Treg, as an inhibitory immune cell, revealed that vaccination with a liposomal form of peptide with/without CpG or Peptide+CpG groups led to a lower number of Tregs compared to control groups (*P*<0.05) (Figure 4J). In addition, there was a significant decrease in IL-10 production by regulatory T cells comparing the control group with the Li-peptide group (*P*<0.01) and those groups with CpG adjuvant (*P*<0.001). Vaccination with peptide also showed a lower titer of IL-10 than the control group (*P*<0.05) (Figure 4K).

*The Frequency of TILs followed by combination therapy*

**Figure 5:**
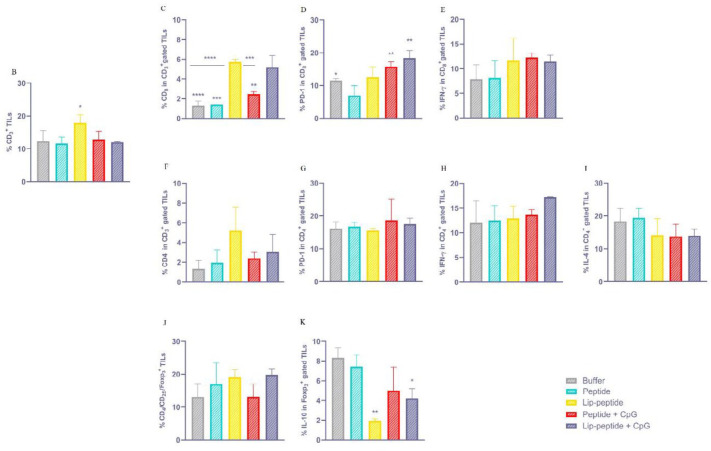
Therapeutic effects of liposomal nanovaccine in combination with anti PD-1 mAb

In analysis of tumor infiltrated lymphocytes (TILs) in tumor, we found a significant difference (*P*<0.05) between the number of infiltrated lymphocytes of peptide and Lip-peptide groups (Figure 5B). By considering CD_8_^+^ TILs, the highest number of TILs was seen in Lip-peptide or Lip-peptide+CpG groups (*P*<0.0001) compared to control groups and peptide group (*P*<0.0001 and *P*<0.001, respectively). Also, these groups were superior than Peptide+CpG group (*P*<0.001 and *P*<0.01) (Figure 5C). 

In TME, both groups consisting of CpG adjuvant had a significantly higher number of PD-1^+^ CD_8_^+^ TILs than the peptide group (*P*<0.01) (Figure 5D). Although there was no significant difference between treatment groups, liposomal or CpG containing groups secreted IFN-γ more than the peptide group (Figure 5E). Furthermore, although there were no differences in the infiltration of antigen-specific CD_4_^+^ PD-1^+^ TILs into TME (Figure 5F & G), these cells were functional, which was confirmed by the secreting IFN-γ (Figure 5H). The same results were seen for IL-4 production (Figure 5I). Formulation of the peptide in liposomal form improved the efficacy of combination therapy through inhibition of IL-10 secretion over the peptide group and even with anti PD-1 monotherapy (*P*<0.01). The liposomal group with CpG also showed a significant decrease compared to anti PD-1 monotherapy (*P*<0.05) (Figure 5K). 

***The frequency of IFN-γ secreting splenocytes and tumor cells***

**Figure 6:**
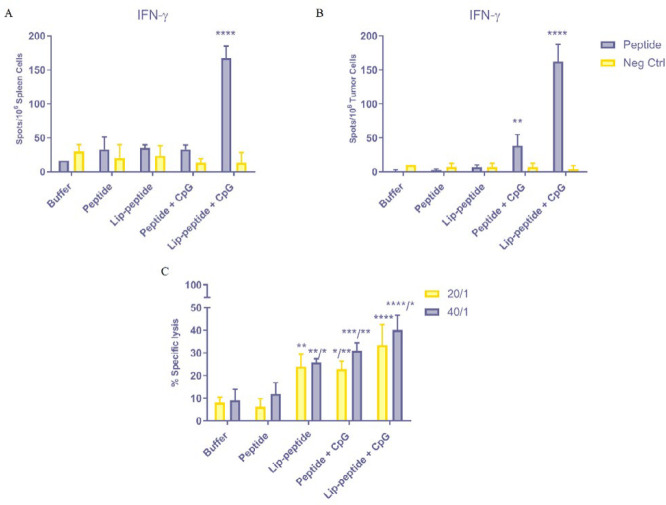
*In vitro* evaluation of the combination therapy efficacy

To assess the stimulatory capacity of different peptide formulation vaccines *in vivo*, weperformed ELISpot analysis of T cells activation *via* quantification of extracellular release of IFN-γ in spleen and TME. As shown in Figure 6A, concomitant administration of CpG with liposomal peptide vaccine significantly (*P*<0.0001) induced T cells activation with a higher release of IFN-γ than other groups in the spleen. A similar trend was observed in the release of IFN-γ by TILs in TME for Lip-peptide+CpG group (*P*<0.0001). Moreover, treatment with Peptide+CpG formulation has also enhanced the functionality of TILs with increased production of IFN-γ over Lip-peptide, peptide or control groups (Figure 6B). This data confirmed that co-administration of CpG adjuvant with therapeutic vaccine formulation could strongly increase the production of IFN-γ *in vivo*.

***Enhanced CTLs activity by combination therapy***

Figure 6C showed the ability of different gp100 peptide formulations in combination with anti PD-1 mAb to elicit cytotoxic activity of antigen-specific CD_8_^+ ^T cells. Vaccination with gp100 conjugated liposomes showed significant specific lysis of target cells compared to the peptide group with 20/1 and 40/1 ratios (*P*<0.01 and* P*<0.05, respectively), and also compared to the control group (*P*<0.01). Treating mice with Peptide+CpG vaccine have led to a significantly higher cytotoxic activity than treating them with just anti PD-1 (*P*<0.05 for 20/1 and *P*<0.001 for 40/1) and free peptide (*P*<0.01 for both ratios). In consistent with the results of IFN-γ production by CD_8_^+^ T cells in figure 4E, concomitant administration of liposomal peptide with CpG adjuvant had a significant role in the killing of target cells in anti PD-1 and peptide groups in both ratios (*P*<0.0001) and also in Lip-peptide group in 40/1 ratio (*P*<0.05). 

***Determination of mRNA expression level following combination therapy***

**Figure 7:**
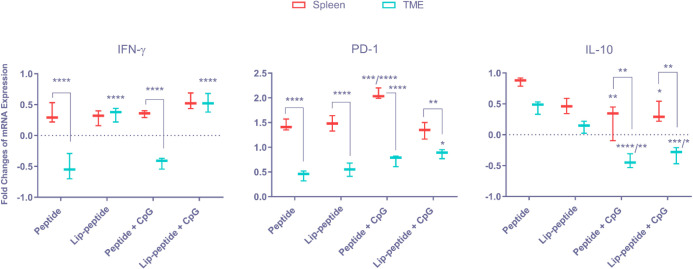
Relative Changes in the mRNA expression followed combination therapy

Besides the flow cytometric and ELISpot assessment of cytokine production, we also performed mRNA expression level analysis *via* quantitative Real-time PCR in both spleen and TME. Results of IFN-γ analysis have shown that there was no significant difference between treatment groups in spleen, but in comparison with IFN-γ expression level in spleen and TME, in peptide groups with and without CpG, the mRNA expression in TME was significantly lower than that of spleen (*P*<0.0001). In contrast, in both liposomal groups (with/without CpG), the mRNA expression was upregulated in TME similar to spleen (*P*>0.05). Also, in TME these two groups significantly increased mRNA expression level over peptide or Peptide+CpG groups with *P*<0.0001, which showed the efficacy of liposomal vaccines in combination with mAb in the infiltration of TILs (Figure 7A). In the analysis of PD-1 expression level, we found increased expression in Peptide+CpG group compared to peptide or Lip-peptide groups (*P*<0.001) and Lip-peptide+CpG group (*P*<0.0001). Comparing the two tissues, the highest expression level was seen in Lip-peptide+CpG group with the lowest difference than in spleen (*P*<0.01). In contrast, a significant (*P*<0.0001) low expression level was seen in other groups. These results also confirmed the efficacy of Lip-peptide+CpG in infiltration of T cells (Figure 7B). Treatment with Peptide+CpG or Lip-peptide+CpG resulted in decreased expression level of IL-10 in spleen compared to peptide group (*P*<0.01 and *P*<0.05, respectively). In TME, the level of IL-10 expression was downregulated significantly in these two groups compared to the peptide group (*P*<0.0001 and *P*<0.01) and Lip-peptide group (*P*<0.001 and *P*<0.05). Comparing spleen with TME, both Peptide+CpG and Lip-peptide+CpG groups decreased more significantly in TME than in spleen (*P*<0.01). In both tissues, even though there was no significant difference (*P*>0.05) between peptide and Lip-peptide groups, Lip-peptide group had a lower level of IL-10 expression than the peptide group (Figure 7C).

***Increased tumor infiltrated lymphocytes (TILs) following combination therapy***

**Figure 8:**
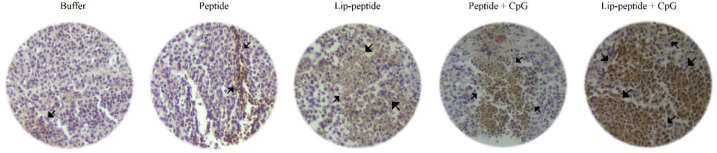
Immunohistochemical analysis of TILs in TME after combination therapy

Immunohistochemical analysis of tumor tissues in different treatment groups also showed the highest infiltration of T cells in the group of mice treated with Lip-peptide+CpG vaccine in combination with anti PD-1 mAb. As were detected in tumor sections, Lip-peptide or Peptide+CpG groups showed a similar level of T cells’ infiltration into tumors (Figure 8).

***Therapeutic efficacy of combination therapy***

**Figure 9:**
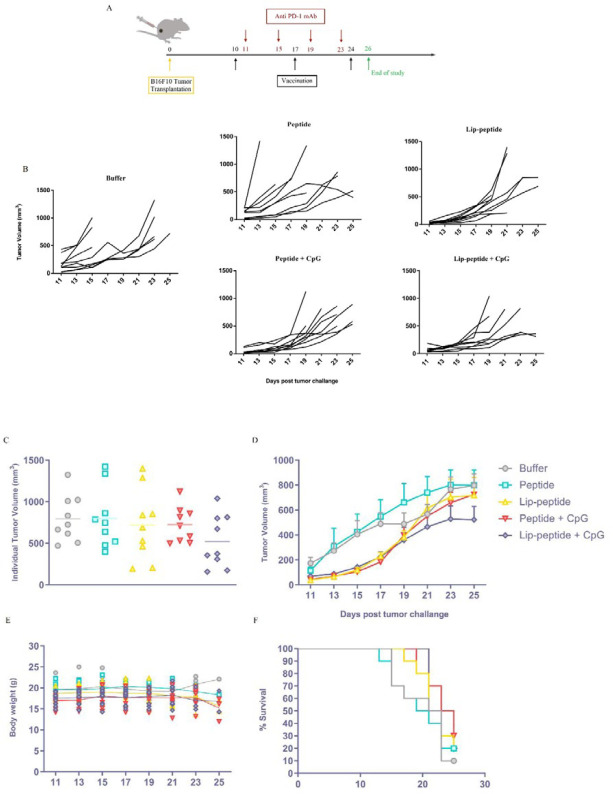
Enhanced antitumor effects in the B16F10 melanoma mouse model followed combination therapy. (A) Experimental schedules of C57BL/6 tumor-bearing mice combination therapy with vaccines and anti-PD-1 monoclonal antibody. Tumor growth measurements started at the same time of first priming, followed every two days and presented as tumor volume (mm3). Individual tumor growth curves of animals (B) and Individual tumor volume of B16F10 bearing mice in the last day of study (C). Overall tumor growth in different groups over time (D). Data represent Mean ± standard error of the mean (SEM); N=9. Monitoring the body weight of mice in each treatment groups (E). B16F10 challenged mice were followed for their survival rate up to 25 days and lack of survival was defined as tumor volume > 1000 mm3 or death (F). Differences in survival were determined by the Kaplan–Meier method and the P-value was calculated by the log-rank (Mantel–Cox) test

The Therapeutic efficacy of different vaccines in combination with anti PD-1 mAb was evaluated using the subcutaneous melanoma mouse model (Figure 9A). The data of tumor growth in Fig 9B show an increased trend in both peptide and control groups with a higher tumor volume compared to other groups (Figure 9C & D). The Tumor growth profile of both Lip-peptide and Peptide+CpG groups have shown a slower tumor growth similar to Lip-peptide+CpG group till day 19 (Figure 9B) but this increased in the following days. Figures 9C & D also shows that these groups similarly have a controlled tumor progression. Vaccination with Lip-peptide+CpG formulation provided the greatest inhibition of tumor progression with mean tumor volumes smaller than Lip-peptide and Peptide+CpG groups (Figures 9C & D). Combination therapy with liposomal (44.65 days) and adjuvant consisting of liposomal forms of peptide (53.68 days) have delayed the onset of tumor growth compared to anti PD-1 monotherapy (Table 3). The Result of body weight showed no significant decrease in liposomal treated groups (Figure 9E), which revealed the safe dose of cationic liposome for *in vivo* study (42). Interestingly, despite the tumor growth in Peptide+CpG group, mice treated with this formulation showed the greatest survival rate with MST of 24 days (Table 3) and 3 mice remained alive till the end of study (Figure 9F). 

**Table 1:** The dose of DOTAP, GP100 peptide and CpG adjuvant administered into the mouse with different formulations. Treatment groups were Peptide+CpG, Lip-peptide, and Lip-peptide+CpG

Treatment groups	Formulation	Particle Size (nm)	PdI	Zeta potential (mV)
Peptide + CpG	GP100 + CpG	166.7 ± 5.42	0.287 ± 0.10	- 6.42 ± 0.00
Lip-peptide	DOTAP: Chol: GP100	231.5 ± 3.04	0.315 ± 0.01	6.15 ± 3.09
Lip-peptide + CpG	DOTAP: Chol: GP100 + CpG	277.7 ± 3.83	0.226 ± 0.06	-3.76 ± 6.14

**Table 2:** The physicochemical characteristics (particle size, polydispersity index (PDI), and zeta potential) of liposomal formulations (Lip-peptide) with or without CpG (N=3; Mean±SD)

Treatment groups	DOTAP dose (nmol/mouse)	GP100 dose (µg/mouse)	CpG dose (µg/mouse)
Peptide + CpG	-	50 µg	14.05 µg
Lip-peptide	265 nmol	50 µg	-
Lip-peptide + CpG	265 nmol	50 µg	14.05 µg

(A) The final product of linking reaction was spotted on silica thin-layer plates besides the free peptide and Maleimide-PEG2000-DSPE, for chromatography in Chloroform: Methanol: Water (45:9:1) mobile phase. (B) Analysis of the final product by High-pressure Liquid Chromatography (HPLC) using Nucleosil C18 column with elution of ACN: Water (70:30) containing 0.1% TFA at 220 nm. The percentage of conjugation was determined according to the extent of unconjugated peptide (with retention of ~ 8 mins)

Immature bone marrow-derived dendritic cells (day 7) were stimulated with gp100 peptide (2.5 μg/ml) and cationic liposome (12 nmol/ml) for 8 hours, the same time used for LPS (1µg/ml). Afterward, DCs were stained for DCs specific marker, CD11c, and then for co-stimulatory molecules CD40, CD80, CD86 and MHC II molecule. For the analysis, DCs were gated for CD11c^+^ cells followed by gating for the selected markers. Immature DCs were analyzed on day 5. The numbers in the dot plot graph represent the percentages of matured DCs

(A) Representative whole-body images of DiD-labeled liposomal vaccine at different time points post-injection. (B) *In vitro* imaging of TDLNs 7- and 21-days post-injection. The quantitative fluorescence intensity of the DiD-labeled liposomal vaccine at the whole body (C) and of the signal emitted from TDLNs (D). The fluorescence intensity scale is displayed on the right side of the images

Immune responses determined with FACS Calibur Cell analyzer and presented in the spleen. (S4A) Representative dot plots demonstrating the gating strategies for flow cytometric analysis of CD_3_^+^ T cells and regulatory T cells. (B) Frequency of CD_3_^+^ cells indicating T cells, (C) Frequency of CD_8_ in CD_3_^+^ gated T cells, (D) Frequency of PD-1 in CD_8_^+^ T cells, (E) Frequency of IFN-γ in CD_8_^+^ T cells. (F) Frequency of CD4 in CD_3_^+^ gated T cells, (G) Frequency of PD-1 in CD_4_^+^ T cells, (H) Frequency of IFN-γ in CD_4_^+^ T cells, (I) Frequency of IL-4 in CD_4_^+^ T cells. (J) Frequency of CD_4_/CD_25_/ Foxp_3 _^+^ T cells, (K) Frequency of IL-10 in Foxp_3_^+^ gated T cells. Results represented as Mean±SD; N=3. * *P<*0.05, **P<0.01, ***P<0.001 and ****P<0.0001 indicated as statistically significant differences

Immune responses followed combination therapy in TME. (S5A) Representative dot plots demonstrating the gating strategies for flow cytometric analysis of CD_3_^+^ T cells and regulatory TILs. (B) Frequency of CD_3_^+^ cells indicating TILs, (C) Frequency of CD_8_ in CD_3_^+^ gated TILs, (D) Frequency of PD-1 in CD_8_^+^ TILs, (E) Frequency of IFN-γ in CD_8_^+^ TILs. (F) Frequency of CD_4_ in CD_3_^+^ gated TILs, (G) Frequency of PD-1 in CD_4_^+^ TILs, (H) Frequency of IFN-γ in CD_4_^+^ TILs, (I) Frequency of IL-4 in CD_4_^+^ TILs. (J) Frequency of CD_4_/CD_25_/Foxp_3_^+^ TILs, (K) Frequency of IL-10 in Foxp_3_^+^ gated TILs. Results represented as Mean±SD; N=3. * *P<*0.05, **P<0.01, ***P<0.001 and ****P<0.0001 indicated as statistically significant differences

Extracellular determination of IFN-γ production by ELIspot using splenocytes and tumor cells (A&B). Cytotoxic activity of CTLs of the treated mice (C). Data represented as Means±SD; N=3. ** *P<*0.01 ***P<0.001 and ****P<0.0001

Expression of IFN-γ (A), PD-1 inhibitory receptor (B) and IL-10 (C) cytokines at peripheral sites – Spleen – and TME of the treated mice. Data were analyzed relative to expression levels of group treated with Buffer in combination with mAb. Data represented as Means±SD; N=3. ** *P<*0.01 ***P<0.001 and ****P<0.0001

Representative images are shown the infiltration of lymphocytes in the tumor sections obtained from mice received anti-PD-1 mAb in combination with different vaccines. Arrows illustrated the TILs that were blocked with anti-PD-1 antibody

**Table 3:** The antitumor efficacy of different therapeutic vaccine formulations including Buffer, Peptide, Lip-peptide, or Peptide+CpG in combination with anti PD-1 mAb in B16F10 tumor bearing mice model (N=9)

Treatment groups	TTE^a^ (Days±SD)	TGD^b^ (%)	MST^c^ (Days)	ILS^d^ (%)
Buffer	19.54 ± 7.75	-	22	-
Peptide	18.97 ± 7.92	-2.89	20	-9.1
Lip-peptide	28.26 ± 13.94	44.65	22	0
Peptide + CpG	24.20 ± 3.36	23.85	24	9.09
Lip-peptide + CpG	30.03 ± 12.34	53.68	21	-4.55

^a^ Time to reach end point; ^b^ Tumor growth delay; ^c^ Median survival time; ^d^ Increased life span.

## Discussion

The generation of a robust, long-lasting and specific immune response is the main goal of cancer immunotherapy (43). Meanwhile, the immunosuppressive condition of TME makes malignant tumors overcome the immune system and tumor invasion, which subsequently leads to the rapid growth of tumors and metastasis (43, 44). One way to overcome this issue is to use the inhibitory receptors that block antibodies (45). In the present study, we used the PD-1 receptor blocking antibody to overcome the immunosuppression of B16F10 melanoma in TME. Despite the promising benefit of anti PD-1 therapy, its efficacy is limited due to the induced resistance in the early phase or during the process of therapy (46). The potential mechanism of PD-1 monoclonal antibodies is through the inhibition of T cell exhaustion and anergy in TME (47). Since PD-1 receptors are mainly expressed on antigen-activated T cells (48), the induction of robust antigen-specific immune responses would enhance the efficacy of anti PD-1 immunotherapy (6). 

The Combination of anti-cancer vaccines with immunotherapy has been used as an appealing therapeutic approach due to its potential in generating potent and durable antigen-specific immune responses (49, 50). To this end, we used the liposomal-peptide vaccine to enhance the immunogenicity of gp100_25-33 _melanoma self-antigen and enlarge the antigen-specific T cells pool. Following that, we investigated its therapeutic effect in combination with anti PD-1 mAb in the mouse model of B16F10 tumor. 

Herein, we described the utility of cationic liposomes (CLs) as an antigen delivery system for *in vivo* immunization in which dendritic cells, as the main antigen presenting cells (APCs), have an important role in initiating antigen-specific T cell responses. Thus, maturation and activation of DCs are the prerequisites for the induction of an efficient immunity (15, 16, 51). As is shown in our results, DOTAP containing liposomes could markedly promote bone marrow-derived dendritic cells (BMDCs) maturation and activation as indicated by the upregulation of CD40, CD80, CD86 and MHC II *in vitro *(15, 52). 

Parallel to the role of positive charge of cationic liposomes in attracting the negatively charged surface of DCs (53), local depot formation at the injection site also improves DCs uptake. This has been exhibited predominantly by cationic liposomes compared to anionic and neutral ones, which would be sufficient for the induction of Th1 immune responses (54, 55). Our findings have demonstrated that vaccination with liposomal-peptide increased the CD_4_^+^ T cells population, as one of the important factors in the mediation of potent antitumor immunity *via* enhancement of cytotoxic T cell activity (56). On the other hand, CLs also enhance cross-presentation by elevation of lysosomal pH, decreasing antigen degradation which leads to cross-presentation (57).

Furthermore, the method through which antigen is associated with the liposomal formulation *via* either covalent or non-covalent conjugation to liposome, encapsulation in or adsorption onto the surface liposome, is one of the main parameters that influences the liposomal-peptide vaccine immunogenicity. Earlier studies have confirmed the efficacy of both encapsulation and conjugation methods in the induction of antigen-specific T cell immunity (58, 59). In contrast to the presented experimental studies, in some cases, it was reported that covalent conjugation of antigen has resulted in improved uptake by phagocytic cells and superior induction of helper and cytotoxic T cell responses. As was reported by Chen and Huang, covalent conjugated E7 into cationic liposome has resulted in 2-fold increase of CTL responses through direct intracellular trafficking of antigen and presenting *via* MHC I molecules (60). This point was also shown in our previous studies that the conjugation of HER2/neu derived P5 peptide to a different liposomal construct was effective as a preventive and therapeutic vaccine in mediating antitumor immunity in TUBO tumor mouse model (61-64). Our findings have also indicated that mice treated with liposome conjugated-peptide had a therapeutic antigen-specific immune response with an increased number of CD_8_^+^ T cells producing IFN-γ compared to peptide and control groups, proving the effective role of covalent conjugation of antigen in CTL induction.

Another influential parameter is the particle size of liposomal cancer vaccine which affects the efficacy of vaccination. It was shown that soluble antigens with a small size of around 10 nm rapidly diffuse out of the lymphatic system, which limits its uptake by APCs, whereas nanoparticles with a larger size, more than 500 nm, will be entrapped within the proteins of extracellular matrix at the injection site (13, 65). Meanwhile, the liposomal-peptide construct with an intermediate size will be either retained at the injection site or drained to the draining lymph nodes, which in both cases, have a chance to be uptaken by APCs, as well as presenting and inducing T cell activation (66). Our *in vivo* analysis of the tracking liposomal construct has proved the proper uptake of covalently conjugated cationic nano-vaccines with the size of 231 nm by DCs and migration from the site of injection to TDLNs. 

The persistent activation of T cell immune responses depends on proper uptake and durable presentation of antigen by DCs. This way, co-administration of adjuvant with antigen would be helpful (67). CpG oligonucleotide (CpG-ODN) adjuvant, as an agonist of TLR9, serves as a danger signal that could enhance the innate immune system (68). To activate an adaptive immune response, the size of exogenous antigen significantly affects the uptake by DCs (69). Co-administration of TLRs with antigen will result in particle formation with a desirable size for uptake by DCs. Moreover, the presence of TLRs leads to an enhanced presentation of antigen, which consequently motivates a potent and durable adaptive immune system (70). Results from earlier studies have shown the efficacy of antigen and CpG-ODN mixture in the induction of Th1 based immunity and tumor rejection (71). We have also shown the efficacy of co-administration of CpG-ODN adjuvant in the enhancement of peptide vaccine immunogenicity in HER2 positive breast cancer model (72-74). In consistent with the mentioned studies, we have found that the mixture of gp100 antigen with CpG-ODN resulted in particle formation with a notable size of 166 nm, which in *in vivo* immunization lead to the significant activation of both antigen-specific CD_4_^+^ and CD_8_^+^ T cells producing IFN-γ. Also, it enhanced the cytotoxic activity of CTLs compared to the peptide group. 

Considering the therapeutic role of adjuvant, since cationic liposome, mainly DOTAP lipid, has an adjuvant property (15, 16), those groups of mice treated with Lip-peptide and Peptide+CpG almost had the same results in the induction of immune responses in spleen and also in overall control of tumor growth. 

Studies of the melanoma mouse model have indicated that subcutaneous administration of nanoparticles with CpG adjuvant had resulted in proper accumulation in draining lymph nodes and was effective as an antitumor immunotherapeutic approach (75, 76). The same results were shown in DOTAP containing cationic liposomes combined with CpG adjuvant as a preventive cancer vaccine in HER2 positive breast cancer model (72, 77). In our study, co-delivery of electro static combined CpG-ODN to gp100 conjugated cationic liposomes displayed a potent antigen-specific mediated immunity in the B16F10 melanoma mouse model. As is shown, the treatment with Lip-peptide+CpG formulation enhanced CTL activation and IFN-γ production and resulted in the highest cytotoxic activity over other treated groups. Treatment with this formulation delayed the tumor growth and eliminated tumor volume up to 500 mm^3^, although tumor eradication was not achieved. 

In combination with anti PD-1 mAb, both peptide and Lip-peptide consisting of CpG-ODN had a significant increase in the number of CD_8_^+^ PD-1^+^ TILs compared to the peptide group. For Lip-peptide and Peptide+CpG groups, the number of these cells were at the same level as were shown in the flow cytometric and immunohistochemical analysis. Considering the efficacy of the combination therapy, in all groups that received the cancer vaccine, the amount of IL-10 production was decreased by regulatory T cells. Overall, the combination of Lip-peptide+CpG vaccine with anti PD-1 mAb led to a significant increase in the number of TILs (as was shown in IHC graph) IFN-γ producing cells. 

## Conclusion

In the present study, we have synthetized a peptide vaccine in liposomal form as a combination therapy with PD-1 blocker antibody. *In vivo* administration of gp100 peptide conjugated to cationic liposome promoted DCs uptake and homing to tumor draining lymph nodes which enhanced its therapeutic efficacy. Co-administration of CpG-ODN with liposomal formulation in combination with anti PD-1 mAb have increased the number of IFN-γ producing CD_8_^+^ PD-1^+^ TILs and their antigen-specific cytotoxic activity, which consequently, inhibited tumor growth progression in an established B16F10 melanoma tumor. Therefore, the combination of cationic liposome conjugated gp100 and CpG-ODN with anti PD-1 monoclonal antibody can be regarded as a potential therapeutic approach for the enhancement of immunotherapy in melanoma. 

## References

[1] Fan Q CZ, Wang C, Liu Z (2018). Toward biomaterials for enhancing immune checkpoint blockade therapy. Adv Funct Mater.

[2] Van der Burg SH, Arens R, Ossendorp F, van Hall T, Melief CJ (2016). Vaccines for established cancer: overcoming the challenges posed by immune evasion. Nat Rev Cancer.

[3] Fridman WH, Pages F, Sautes-Fridman C, Galon J (2012). The immune contexture in human tumours: impact on clinical outcome. Nat Rev Cancer.

[4] Spranger S, Koblish HK, Horton B, Scherle PA, Newton R, Gajewski TF (2014). Mechanism of tumor rejection with doublets of CTLA-4, PD-1/PD-L1, or IDO blockade involves restored IL-2 production and proliferation of CD8+ T cells directly within the tumor microenvironment. J Immunother Cancer.

[5] Gajewski TF (2015). The next hurdle in cancer immunotherapy: Overcoming the non-T-cell-inflamed tumor microenvironment. Semin Oncol.

[6] Tumeh PC, Harview CL, Yearley JH, Shintaku IP, Taylor EJ, Robert L (2014). PD-1 blockade induces responses by inhibiting adaptive immune resistance. Nature.

[7] Chowdhury PS, Chamoto K, Honjo T (2018). Combination therapy strategies for improving PD-1 blockade efficacy: a new era in cancer immunotherapy. J Intern Med.

[8] Byrne KT, Cote AL, Zhang P, Steinberg SM, Guo Y, Allie R (2011). Autoimmune melanocyte destruction is required for robust CD8+ memory T cell responses to mouse melanoma. J Clin Invest.

[9] Dougan M DG (2009). Immune therapy for cancer. Annu Rev Immunol.

[10] Smith DM, Simon JK, Baker JR, Jr (2013). Applications of nanotechnology for immunology. Nat Rev Immunol.

[11] Korsholm KS, Andersen PL, Christensen D (2012). Cationic liposomal vaccine adjuvants in animal challenge models: overview and current clinical status. Expert Rev Vaccines.

[12] Xiang SD, Scholzen A, Minigo G, David C, Apostolopoulos V, Mottram PL (2006). Pathogen recognition and development of particulate vaccines: does size matter?. Methods.

[13] Oussoren C, Zuidema J, Crommelin DJ, Storm G (1997). Lymphatic uptake and biodistribution of liposomes after subcutaneous injection Influence of liposomal size, lipid compostion and lipid dose. Biochim Biophys Acta.

[14] Christensen D, Korsholm KS, Rosenkrands I, Lindenstrom T, Andersen P, Agger EM (2007). Cationic liposomes as vaccine adjuvants. Expert Rev Vaccines.

[15] Vangasseri DP, Cui Z, Chen W, Hokey DA, Falo LD Jr, Huang L (2006). Immunostimulation of dendritic cells by cationic liposomes. Mol Membr Biol.

[16] Yan W, Chen W, Huang L (2007). Mechanism of adjuvant activity of cationic liposome: phosphorylation of a MAP kinase, ERK and induction of chemokines. Mol Immunol.

[17] Steinhagen F, Kinjo T, Bode C, Klinman DM (2011). TLR-based immune adjuvants. Vaccine.

[18] Khazanov E, Simberg D, Barenholz Y (2006). Lipoplexes prepared from cationic liposomes and mammalian DNA induce CpG-independent, direct cytotoxic effects in cell cultures and in mice. J Gene Med.

[19] Whitmore MM, Li S, Falo L Jr, Huang L (2001). Systemic administration of LPD prepared with CpG oligonucleotides inhibits the growth of established pulmonary metastases by stimulating innate and acquired antitumor immune responses. Cancer Immunol Immunother.

[20] Kaczanowska S, Joseph AM, Davila E (2013). TLR agonists: our best frenemy in cancer immunotherapy. J Leukoc Biol.

[21] Latz E, Schoenemeyer A, Visintin A, Fitzgerald KA, Monks BG, Knetter CF (2004). TLR9 signals after translocating from the ER to CpG DNA in the lysosome. Nat Immunol.

[22] Klinman DM, Currie D, Gursel I, Verthelyi D (2004). Use of CpG oligodeoxynucleotides as immune adjuvants. Immunol Rev.

[23] Krieg AM (2002). CpG motifs in bacterial DNA and their immune effects. Annu Rev Immunol.

[24] Puangpetch A, Anderson R, Huang YY, Sermswan RW, Chaicumpa W, Sirisinha S (2012). Cationic liposomes extend the immunostimulatory effect of CpG oligodeoxynucleotide against Burkholderia pseudomallei infection in BALB/c mice. Clin Vaccine Immunol.

[25] Davila E, Kennedy R, Celis E (2003). Generation of antitumor immunity by cytotoxic T lymphocyte epitope peptide vaccination, CpG-oligodeoxynucleotide adjuvant, and CTLA-4 blockade. Cancer Res.

[26] Mangsbo SM, Sandin LC, Anger K, Korman AJ, Loskog A, Totterman TH (2010). Enhanced tumor eradication by combining CTLA-4 or PD-1 blockade with CpG therapy. J Immunother.

[27] Wang S, Campos J, Gallotta M, Gong M, Crain C, Naik E (2016). Intratumoral injection of a CpG oligonucleotide reverts resistance to PD-1 blockade by expanding multifunctional CD8+ T cells. Proc Natl Acad Sci U S A.

[28] Gholizadeh Z, Tavakkol-Afshari J, Nikpoor AR, Jalali SA, Jaafari MR (2018). Enhanced immune response induced by P5 HER2/neu-derived peptide-pulsed dendritic cells as a preventive cancer vaccine. J Cell Mol Med.

[29] Pappalardo F, Pennisi M, Ricupito A, Topputo F, Bellone M (2014). Induction of T-cell memory by a dendritic cell vaccine: a computational model. Bioinformatics.

[30] Filion MC, Phillips NC (1997). Toxicity and immunomodulatory activity of liposomal vectors formulated with cationic lipids toward immune effector cells. Biochim Biophys Acta.

[31] Moynihan KD, Opel CF, Szeto GL, Tzeng A, Zhu EF, Engreitz JM (2016). Eradication of large established tumors in mice by combination immunotherapy that engages innate and adaptive immune responses. Nat Med.

[32] Fu J, Malm IJ, Kadayakkara DK, Levitsky H, Pardoll D, Kim YJ (2014). Preclinical evidence that PD1 blockade cooperates with cancer vaccine TEGVAX to elicit regression of established tumors. Cancer Res.

[33] Blake SJ, Ching AL, Kenna TJ, Galea R, Large J, Yagita H (2015). Blockade of PD-1/PD-L1 promotes adoptive T-cell immunotherapy in a tolerogenic environment. PloS one.

[34] Shindo Y, Yoshimura K, Kuramasu A, Watanabe Y, Ito H, Kondo T (2015). Combination immunotherapy with 4-1BB activation and PD-1 blockade enhances antitumor efficacy in a mouse model of subcutaneous tumor. Anticancer Res.

[35] Danciu C, Oprean C, Coricovac DE, Andreea C, Cimpean A, Radeke H (2015). Behaviour of four different B16 murine melanoma cell sublines: C57BL/6J skin. Int J Exp Pathol.

[36] Satterlee AB, Rojas JD, Dayton PA, Huang L (2017). Enhancing Nanoparticle Accumulation and Retention in Desmoplastic Tumors via Vascular Disruption for Internal Radiation Therapy. Theranostics.

[37] Kalli F, Machiorlatti R, Battaglia F, Parodi A, Conteduca G, Ferrera F (2013). Comparative analysis of cancer vaccine settings for the selection of an effective protocol in mice. J Transl Med.

[38] Schluep T, Hwang J, Cheng J, Heidel JD, Bartlett DW, Hollister B (2006). Preclinical efficacy of the camptothecin-polymer conjugate IT-101 in multiple cancer models. Clin Cancer Res.

[39] Prezado Y, Sarun S, Gil S, Deman P, Bouchet A, Le Duc G (2012). Increase of lifespan for glioma-bearing rats by using minibeam radiation therapy. J Synchrotron Radiat.

[40] Mishina H, Watanabe K, Tamaru S, Watanabe Y, Fujioka D, Takahashi S (2014). Lack of phospholipase A2 receptor increases susceptibility to cardiac rupture after myocardial infarction. Circ Res.

[41] Razazan A, Behravan J, Arab A, Barati N, Arabi L, Gholizadeh Z (2017). Conjugated nanoliposome with the HER2/neu-derived peptide GP2 as an effective vaccine against breast cancer in mice xenograft model. PloS one.

[42] Vasievich EA, Chen W, Huang L (2011). Enantiospecific adjuvant activity of cationic lipid DOTAP in cancer vaccine. Cancer Immunol Immunother.

[43] Zeng Q, Jiang H, Wang T, Zhang Z, Gong T, Sun X (2015). Cationic micelle delivery of Trp2 peptide for efficient lymphatic draining and enhanced cytotoxic T-lymphocyte responses. J Control Release.

[44] Li SY, Liu Y, Xu CF, Shen S, Sun R, Du XJ (2016). Restoring anti-tumor functions of T cells via nanoparticle-mediated immune checkpoint modulation. J Control Release.

[45] Chen S, Lee LF, Fisher TS, Jessen B, Elliott M, Evering W (2015). Combination of 4-1BB agonist and PD-1 antagonist promotes antitumor effector/memory CD8 T cells in a poorly immunogenic tumor model. Cancer Immunol Res.

[46] Gide TN, Wilmott JS, Scolyer RA, Long GV (2018). Primary and Acquired Resistance to Immune Checkpoint Inhibitors in Metastatic Melanoma. Clin Cancer Res.

[47] McDermott DF, Atkins MB (2013). PD-1 as a potential target in cancer therapy. Cancer Med.

[48] Topalian SL, Drake CG, Pardoll DM (2012). Targeting the PD-1/B7-H1(PD-L1) pathway to activate anti-tumor immunity. Curr Opin Immunol.

[49] Mahoney KM, Rennert PD, Freeman GJ (2015). Combination cancer immunotherapy and new immunomodulatory targets. Nat Rev Drug Discov.

[50] Chen DS, Mellman I (2017). Elements of cancer immunity and the cancer-immune set point. Nature.

[51] Chen W, Yan W, Huang L (2008). A simple but effective cancer vaccine consisting of an antigen and a cationic lipid. Cancer Immunol Immunother.

[52] Saremi SS, Shahryari M, Ghoorchian R, Eshaghian H, Jalali SA, Nikpoor AR (2018). The role of nanoliposome bilayer composition containing soluble leishmania antigen on maturation and activation of dendritic cells. Iran J Basic Med Sci.

[53] Foged C, Arigita C, Sundblad A, Jiskoot W, Storm G, Frokjaer S (2004). Interaction of dendritic cells with antigen-containing liposomes: effect of bilayer composition. Vaccine.

[54] Henriksen-Lacey M, Christensen D, Bramwell VW, Lindenstrom T, Agger EM, Andersen P (2010). Liposomal cationic charge and antigen adsorption are important properties for the efficient deposition of antigen at the injection site and ability of the vaccine to induce a CMI response. J Control Release.

[55] Henriksen-Lacey M, Christensen D, Bramwell VW, Lindenstrom T, Agger EM, Andersen P (2011). Comparison of the depot effect and immunogenicity of liposomes based on dimethyldioctadecylammonium (DDA), 3beta-[N-(N’,N’-Dimethylaminoethane)carbomyl] cholesterol (DC-Chol), and 1,2-Dioleoyl-3-trimethylammonium propane (DOTAP): prolonged liposome retention mediates stronger Th1 responses. Mol Pharm.

[56] Knutson KL, Disis ML (2005). Tumor antigen-specific T helper cells in cancer immunity and immunotherapy. Cancer Immunol Immunother.

[57] Gao J, Ochyl LJ, Yang E, Moon JJ (2017). Cationic liposomes promote antigen cross-presentation in dendritic cells by alkalizing the lysosomal pH and limiting the degradation of antigens. International journal of nanomedicine.

[58] Shahum E, Therien HM (1995). Liposomal adjuvanticity: effect of encapsulation and surface-linkage on antibody production and proliferative response. Int J Immunopharmacol.

[59] Shariat S, Badiee A, Amir Jalali S, Mansourian M, Alireza Mortazavi S, Reza Jaafari M (2015). Preparation and characterization of different liposomal formulations containing P5 HER2/neu-derived peptide and evaluation of their immunological responses and antitumor effects. Iran J Basic Med Sci.

[60] Chen W, Huang L (2008). Induction of cytotoxic T-lymphocytes and antitumor activity by a liposomal lipopeptide vaccine. Mol Pharm.

[61] Shariat S, Badiee A, Jalali SA, Mansourian M, Yazdani M, Mortazavi SA (2014). P5 HER2/neu-derived peptide conjugated to liposomes containing MPL adjuvant as an effective prophylactic vaccine formulation for breast cancer. Cancer Lett.

[62] Rastakhiz S, Yazdani M, Shariat S, Arab A, Momtazi-Borojeni AA, Barati N (2018). Preparation of nanoliposomes linked to HER2/neu-derived (P5) peptide containing MPL adjuvant as vaccine against breast cancer. J Cell Biochem.

[63] Farzad N, Barati N, Momtazi-Borojeni AA, Yazdani M, Arab A, Razazan A (2019). P435 HER2/neu-derived peptide conjugated to liposomes containing DOPE as an effective prophylactic vaccine formulation for breast cancer. Artif Cells Nanomed Biotechnol.

[64] Zamani P, Navashenaq JG, Nikpoor AR, Hatamipour M, Oskuee RK, Badiee A (2019). MPL nano-liposomal vaccine containing P5 HER2/neu-derived peptide pulsed PADRE as an effective vaccine in a mice TUBO model of breast cancer. J Control Release.

[65] Irvine DJ, Swartz MA, Szeto GL (2013). Engineering synthetic vaccines using cues from natural immunity. Nat Mater.

[66] Oyewumi MO, Kumar A, Cui Z (2010). Nano-microparticles as immune adjuvants: correlating particle sizes and the resultant immune responses. Expert Rev Vaccines.

[67] Joshi MD, Unger WJ, Storm G, van Kooyk Y, Mastrobattista E (2012). Targeting tumor antigens to dendritic cells using particulate carriers. J Control Release.

[68] Bauer M, Redecke V, Ellwart JW, Scherer B, Kremer JP, Wagner H (2001). Bacterial CpG-DNA triggers activation and maturation of human CD11c-, CD123+ dendritic cells. J Immunol.

[69] Guermonprez P, Valladeau J, Zitvogel L, Thery C, Amigorena S (2002). Antigen presentation and T cell stimulation by dendritic cells. Annu Rev Immunol.

[70] Dresch C, Leverrier Y, Marvel J, Shortman K (2012). Development of antigen cross-presentation capacity in dendritic cells. Trends Immunol.

[71] Brazolot Millan CL, Weeratna R, Krieg AM, Siegrist CA, Davis HL (1998). CpG DNA can induce strong Th1 humoral and cell-mediated immune responses against hepatitis B surface antigen in young mice. Proc Natl Acad Sci U S A.

[72] Jalali SA, Sankian M, Tavakkol-Afshari J, Jaafari MR (2012). Induction of tumor-specific immunity by multi-epitope rat HER2/neu-derived peptides encapsulated in LPD Nanoparticles. Nanomedicine.

[73] Ghaffari-Nazari H, Tavakkol-Afshari J, Jaafari MR, Tahaghoghi-Hajghorbani S, Masoumi E, Jalali SA (2015). Improving multi-epitope long peptide vaccine potency by using a strategy that enhances CD4+ T help in BALB/c Mice. PloS one.

[74] Tahaghoghi-Hajghorbani S, Tavakkol-Afshari J, Jaafari MR, Ghaffari-Nazari H, Masoumi E, Jalali SA (2017). Improved immunogenicity against a Her2/neu-Derived peptide by employment of a Pan HLA DR-Binding epitope and CpG in a BALB/c mice model. Anticancer Agents Med Chem.

[75] Jeanbart L, Ballester M, de Titta A, Corthesy P, Romero P, Hubbell JA (2014). Enhancing efficacy of anticancer vaccines by targeted delivery to tumor-draining lymph nodes. Cancer Immunol Res.

[76] Xu Z, Ramishetti S, Tseng YC, Guo S, Wang Y, Huang L (2013). Multifunctional nanoparticles co-delivering Trp2 peptide and CpG adjuvant induce potent cytotoxic T-lymphocyte response against melanoma and its lung metastasis. J Control Release.

[77] Mansourian M, Badiee A, Jalali SA, Shariat S, Yazdani M, Amin M (2014). Effective induction of anti-tumor immunity using p5 HER-2/neu derived peptide encapsulated in fusogenic DOTAP cationic liposomes co-administrated with CpG-ODN. Immunol Lett.

